# Sensing Danger After Reperfusion: TLR7 as a Driver of Myocardial Injury

**DOI:** 10.1016/j.jacbts.2026.101657

**Published:** 2026-09-28

**Authors:** Cong Luo, Matthew DeBerge, Holger Eltzschig

**Affiliations:** Department of Anesthesiology, Critical Care and Pain Medicine, McGovern Medical School, University of Texas Health Science Center at Houston, Houston, Texas, USA

**Keywords:** integrin subunit alpha M, myocardial ischemia-reperfusion injury, toll-like receptor 7

## Abstract

**Figure:**
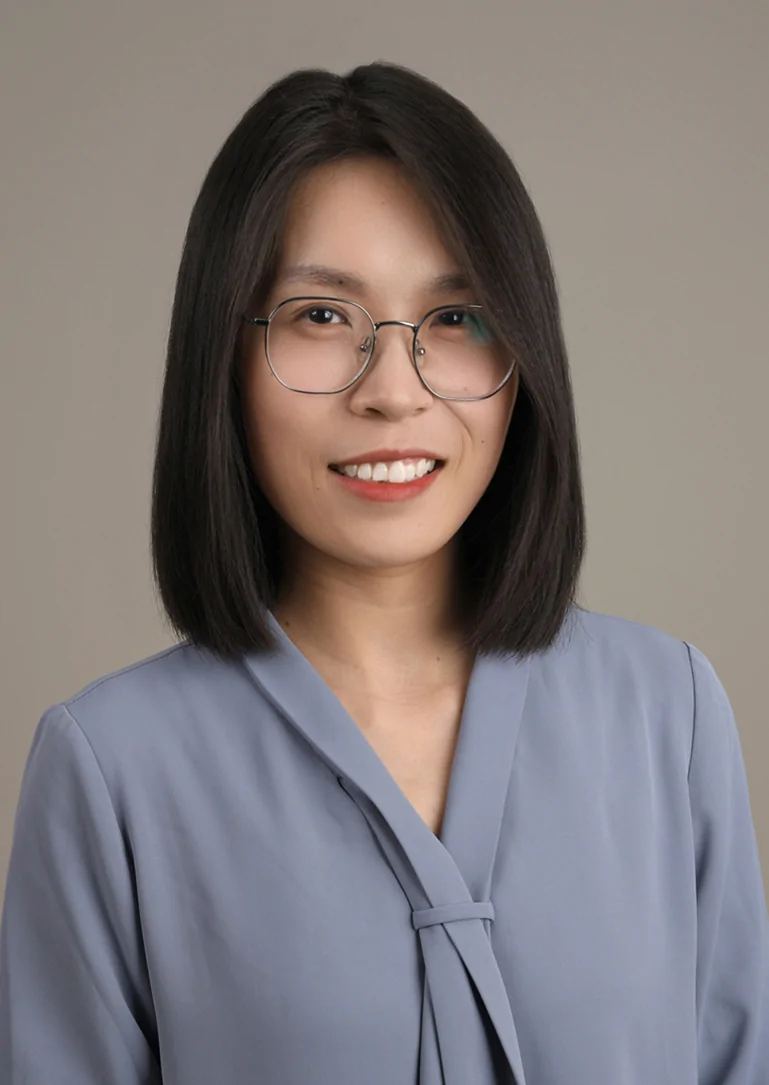
Cong Luo

**Figure:**
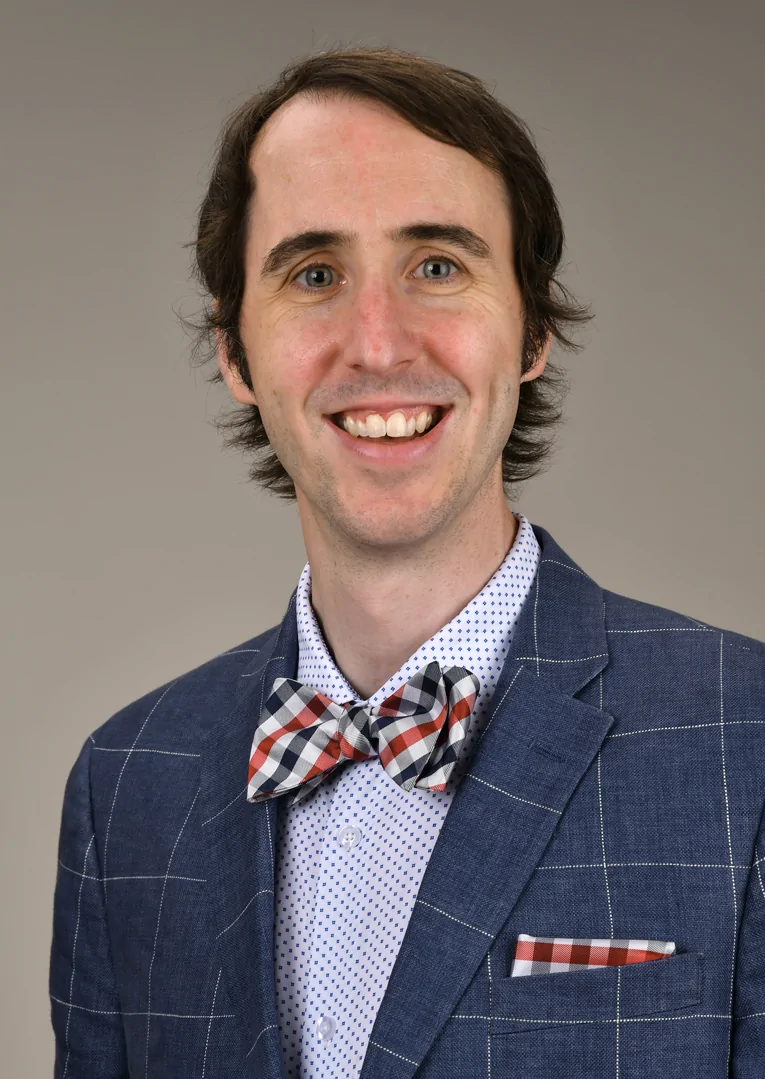
Matthew DeBerge

**Figure:**
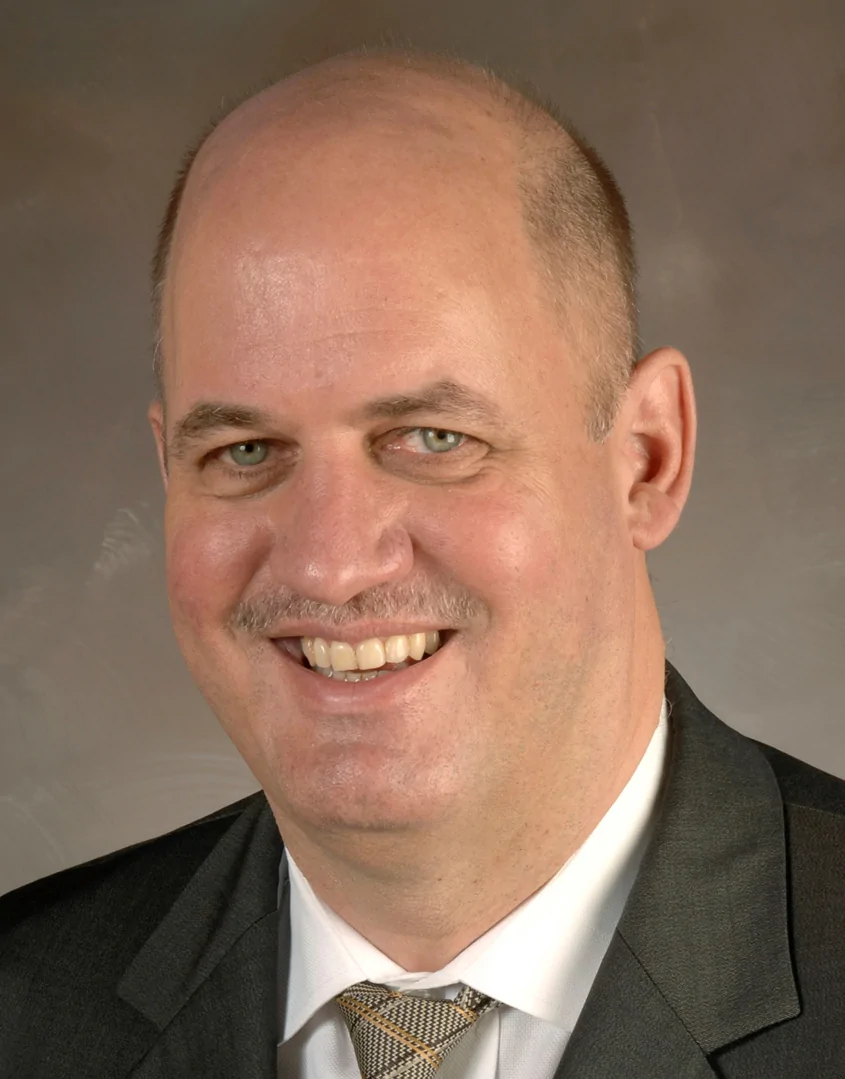
Holger Eltzschig

Cong Luo

Matthew DeBerge

Holger Eltzschig

Primary percutaneous coronary intervention (PCI) has transformed the treatment of ST-segment elevation myocardial infarction (STEMI). Yet despite successful reperfusion, many patients sustain additional myocardial injury that contributes to adverse ventricular remodeling, heart failure, and long-term mortality. Reperfusion is also central to myocardial injury after noncardiac surgery,^1^ in which perioperative ischemic events followed by restoration of blood flow contribute to myocardial damage detected by elevated troponin levels. Thus, reperfusion injury remains one of the most persistent unmet challenges in cardiovascular medicine.^2^ Although numerous therapies have reduced infarct size in experimental models, few have demonstrated clinical benefit. As a result, attention has increasingly shifted from individual inflammatory mediators toward upstream pathways that coordinate the tissue response to myocardial ischemia-reperfusion injury.

Li et al^3^ identify Toll-like receptor 7 (TLR7) as a critical regulator and potential therapeutic target of myocardial ischemia-reperfusion injury. The study begins with a provocative observation: plasma small RNA sequencing revealed a distinct repertoire of circulating RNAs following myocardial ischemia-reperfusion injury. Although traditionally viewed as biomarkers of tissue injury, endogenous nucleic acids can actively regulate inflammatory responses and disease progression.^4^ Li et al^3^ therefore asked whether the tissue-derived multiple micro-RNAs released after myocardial injury function as danger signals that amplify postischemic inflammation. This concept fits within a broader framework in which endogenous stress-response pathways actively determine the balance between injury and repair. Hypoxia-responsive signaling networks, for example, coordinate adaptive responses to oxygen deprivation and modulate inflammatory activation across multiple organ systems.^5^ In the present study, the investigators reasoned that endogenous RNA sensing represents another component of this injury-response circuitry. Focusing on TLR7, an endosomal receptor classically associated with sensing viral single-stranded RNA, they demonstrate through complementary genetic, pharmacologic, transcriptomic, and translational approaches that endogenous RNA sensing promotes endothelial-leukocyte crosstalk, enhances integrin subunit alpha M (ITGAM; also known as CD11b, a well-known pan marker of myeloid cells)–dependent inflammation, and exacerbates infarct expansion following ischemia-reperfusion injury. Supporting a causal role for this pathway, both genetic deletion of TLR7 and pharmacologic inhibition with a clinically investigational oral medication for autoimmune disease, enpatoran, reduced infarct size and preserved cardiac function following ischemia-reperfusion injury. Therefore, Li et al's^3^ findings identified the TLR7-ITGAM axis as a potentially actionable therapeutic pathway, and TLR7 might act as a convergent signaling node for communication between the myocardium and the immune system.

A major strength of the study is the integration of single-nucleus RNA sequencing to define cellular responses within the reperfused myocardium. These analyses revealed expansion of inflammatory myeloid populations alongside endothelial subsets enriched for leukocyte recruitment and adhesion pathways, reinforcing an increasingly appreciated principle in cardiovascular immunology: inflammatory injury rarely arises from a single pathogenic cell type but instead reflects coordinated communication between immune and stromal compartments.^6^ Within this multicellular inflammatory landscape, one of the most striking observations was the robust induction of *Itgam* across multiple cellular populations. ITGAM emerged as a shared inflammatory signature within cardiac leukocytes, endothelial cells, and circulating monocytes. Li et al^3^ demonstrated that TLR7 inhibition suppresses ITGAM induction following myocardial ischemia-reperfusion injury and further showed that TLR7 directly induces *Itgam* expression in macrophages and endothelial cells. Microfluidic adhesion assays further showed that ITGAM enhances leukocyte–endothelial interactions, while analyses of patients with STEMI undergoing PCI confirmed an expansion of circulating ITGAM-positive monocytes. Together, these findings reveal that innate immune sensing drives a coordinated inflammatory program across vascular and immune compartments and establish the TLR7-ITGAM axis as a key mechanistic link between endogenous RNA sensing and leukocyte recruitment.

At first glance, these findings may suggest that ITGAM itself represents an attractive therapeutic target. However, caution is warranted. The history of anti-inflammatory therapy in myocardial infarction is filled with examples of compelling experimental biology that failed to translate into clinical benefit. In particular, strategies targeting leukocyte recruitment, adhesion molecules, and integrin-dependent trafficking have yielded disappointing results despite strong mechanistic rationale and robust preclinical efficacy.^7^ Inflammatory pathways often exert context-dependent functions, contributing to both tissue injury and tissue repair. Broad suppression of leukocyte recruitment therefore risks disrupting reparative responses required for optimal infarct healing and scar formation. Viewed through this lens, the most important contribution of the present study may not be the identification of ITGAM itself, but rather the discovery of an upstream pathway that organizes the adhesive phenotype, revealing a mechanism that coordinates endothelial and myeloid activation before recruitment occurs. TLR7 activation increased ITGAM expression across multiple cellular compartments, promoted endothelial activation, and enhanced leukocyte adhesion, suggesting that the therapeutic opportunity may lie in preventing the inflammatory programming that drives recruitment rather than blocking recruitment itself, which might optimize the infiltrating cell compartment to balance inflammation and repair.

This distinction may prove critical for translation, as it shifts the therapeutic focus toward targeting an upstream sensor of tissue injury rather than downstream antiadhesion mechanisms. By detecting endogenous RNA released from damaged myocardium, TLR7 initiates coordinated inflammatory responses involving endothelial cells, circulating monocytes, and tissue leukocytes. Pharmacologic inhibition attenuated multiple components of this cascade simultaneously, suggesting a broader mechanism of action than direct adhesion blockade alone. Similar principles have been observed in hypoxia-responsive adenosine signaling, where stress sensing in one cell type influences multicellular responses across tissues.^8^ The present study suggests that endogenous RNA sensing through TLR7 may serve a comparable role in reperfused myocardium, functioning upstream of inflammatory programs that drive endothelial activation and leukocyte recruitment. Rather than targeting a single downstream effector, modulation of these sensing pathways has the potential to reshape entire injury-response networks. The availability of enpatoran further enhances the translational appeal of this strategy; TLR7 inhibitors have already advanced to human clinical studies and have demonstrated a favorable safety and tolerability profile,^9^ although efficacy in cardiovascular disease remains untested. Nevertheless, important questions remain. Whether TLR7 inhibition retains efficacy when administered after reperfusion, how it influences long-term infarct healing, and whether similar benefits will be observed in patients with common cardiometabolic comorbidities remain unknown. As with all immunomodulatory approaches, balancing suppression of injurious inflammation against preservation of tissue repair will be essential.

As PCI has largely solved the problem of epicardial coronary occlusion, a remaining challenge in caring for patients with STEMI and myocardial injury after noncardiac surgery is limiting the injury that follows reperfusion. More broadly, perioperative organ injury remains a major driver of morbidity and mortality across cardiovascular and perioperative medicine, with ischemia-reperfusion injury representing a principal underlying mechanism,^1^ highlighting the need for therapies that target fundamental mechanisms of reperfusion-tissue injury. Recent efforts have focused on modulating endogenous stress-response pathways during reperfusion, including hypoxia signaling, innate immune response, resolution of inflammation and promotion of tissue repair, adenosine signaling, metabolic adaptation, and circadian regulation,^5^^,^^10^ with Li et al^3^ now implicating endogenous RNA sensing through TLR7 as an additional regulator (Figure 1). Whether TLR7 inhibition ultimately succeeds where previous anti-inflammatory strategies have failed remains uncertain. Nevertheless, the study provides an important conceptual advance. Rather than targeting a single inflammatory mediator or leukocyte subset, it identifies an upstream injury-sensing pathway that coordinates endothelial activation, immune cell recruitment, and infarct expansion, reinforcing a broader principle emerging in the field that effective cardioprotection may require targeting early adaptive responses to tissue injury rather than isolated downstream inflammatory effectors. Taken together, these findings underscore the continued need for innovative strategies that render the myocardium more resistant to ischemia-reperfusion injury by modulating innate and adaptive immune responses within hypoxic tissues.

**Figure 1:**
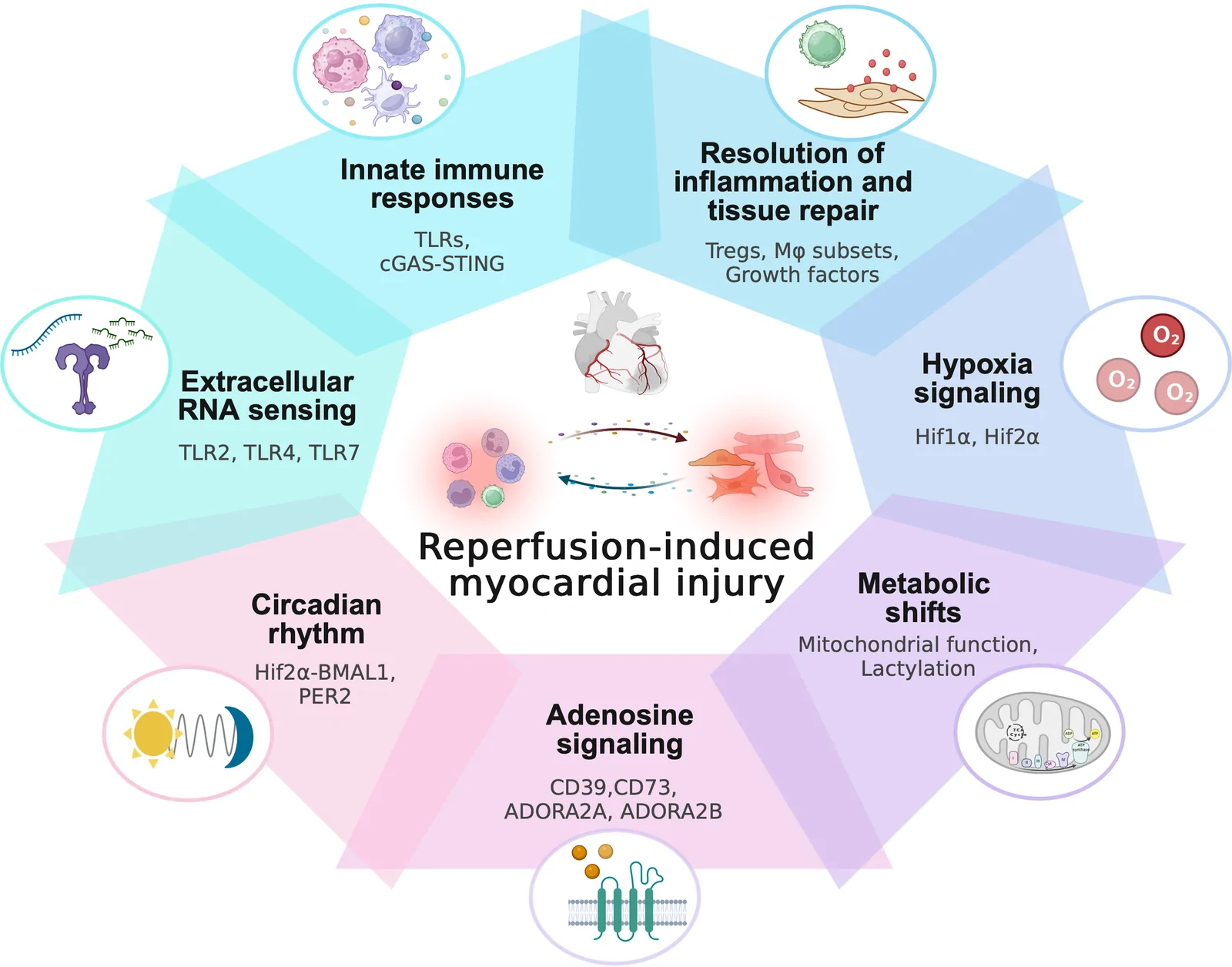
Representative Pathways Involved in Dampening Reperfusion-Induced Myocardial Injury Despite major advances in coronary revascularization and intravascular interventions, reperfusion injury remains a persistent unmet challenge in cardiovascular medicine and perioperative organ protection. Recent efforts have focused on targeting endogenous stress-response and tissue-adaptive pathways to mitigate myocardial ischemia-reperfusion injury. These include modulation of hypoxia signaling (hypoxia-inducible factor 1α [HIF1α] and HIF2α), adenosine signaling, metabolic adaptation, circadian regulation, innate immune responses, inflammatory resolution and tissue repair, and extracellular nucleic acid sensing pathways. Emerging evidence suggests that these pathways collectively coordinate endothelial activation, leukocyte recruitment, cellular metabolism, and tissue repair programs, highlighting multiple opportunities to enhance endogenous cardioprotective mechanisms and improve outcomes following reperfusion. This figure was created using BioRender.com. ADORA2A = adenosine A2A receptor; ADORA2B = adenosine A2B receptor; BMAL1 = brain and muscle ARNT-like 1; cGAS = cyclic guanosine monophosphate–adenosine monophosphate synthase; Mϕ = macrophage; PER2 = period circadian regulator 2; STING = stimulator of interferon genes; TLR = Toll-like receptor; Tregs = regulatory T cells.

## Funding Support and Author Disclosures

Dr Eltzschig is supported by National Institutes of Health grants R01HL165748, R01HL169519, R35HL177402, T32GM135118, and R24HL180372. Dr Eltzschig has a sponsored contract through Akebia Therapeutics (vadadustat for the prevention and treatment ARDS due to suspected aspiration, pathogen-associated pneumonia, or sepsis). All other authors have reported that they have no relationships relevant to the contents of this paper to disclose.
